# Handshake Sense Multiple Access Control for Cognitive Radio-Based IoT Networks

**DOI:** 10.3390/s19020241

**Published:** 2019-01-10

**Authors:** Muhammad Shafiq, Maqbool Ahmad, Muhammad Khalil Afzal, Amjad Ali, Azeem Irshad, Jin-Ghoo Choi

**Affiliations:** 1 Department of Information and Communication Engineering, Yeungnam University, Gyeongsan 38541, Korea; shafiq.pu@gmail.com; 2 Department of Digital Convergence Business, Yeungnam University, Gyeongsan 38541, Korea; maqbool.pu@gmail.com; 3 Department of Computer Science, COMSATS University Islamabad, Wah Campus, Wah Cantt 47040, Pakistan; khalilafzal@ciitwah.edu.pk; 4 Department of Computer Science, COMSATS University Islamabad, Lahore Campus, Lahore 54590, Pakistan; amjad.ali@cuilahore.edu.pk; 5 Department of Computer Science & Software Engineering, International Islamic University, Islamabad 44000, Pakistan; irshadazeem2@gmail.com

**Keywords:** carrier sensing, CSMA/CA, dynamic spectrum access, IoT, spectrum sensing

## Abstract

Internet-of-Things (IoT) enabling technologies such as ZigBee, WiFi, 6LowPAN, RFID, Machine-to-Machine, LTE-Advanced, etc. depend on the license-free Industrial Scientific and Medical (ISM) bands for the Internet. The proliferation of IoT devices is not only anticipated to create a huge amount of congestion in the near future, but even now the unlicensed spectrum is not enough in the ISM bands. Towards this end, Cognitive Radio (CR) technology can resolve the spectrum shortage issue since CR users can opportunistically exploit white spaces in licensed channels of the adjacent wireless systems. In CR networks, it is critical to coordinate spectrum access among Secondary Users (SUs) while protecting priority rights of Primary Users (PUs). Therein, SUs need to take good care of hidden PUs in order to avoid harmful interference. Further, a densely deployed CR network can compromise spectrum sensing quality and certainty of the results when a large number of SUs contends to access the same channel. Therefore, based on the vulnerable sensing results, SUs can cause interference to the PUs. In this paper, we first investigate the leading issues and then propose a novel Handshake Sense Multiple Access with Collision Avoidance (HSMA/CA) protocol for CR-based IoT networks. Our proposed HSMA/CA scheme resolves hidden primary terminal problem and maintains sufficient priority rights to PUs in a densely distributed network. In addition, we optimize the spectrum sensing period to maximize the system performance by maintaining peculiarities in the sensing operation like false alarm and misdetection. To evaluate the performance of HSMA/CA, we have analyzed the protocol through the Markov chain model in terms of throughput and verify its accuracy by simulations. Simulation results show that our scheme is suitable for non-collaborative densely deployed CR-based IoT networks.

## 1. Introduction

**Figure 1:**
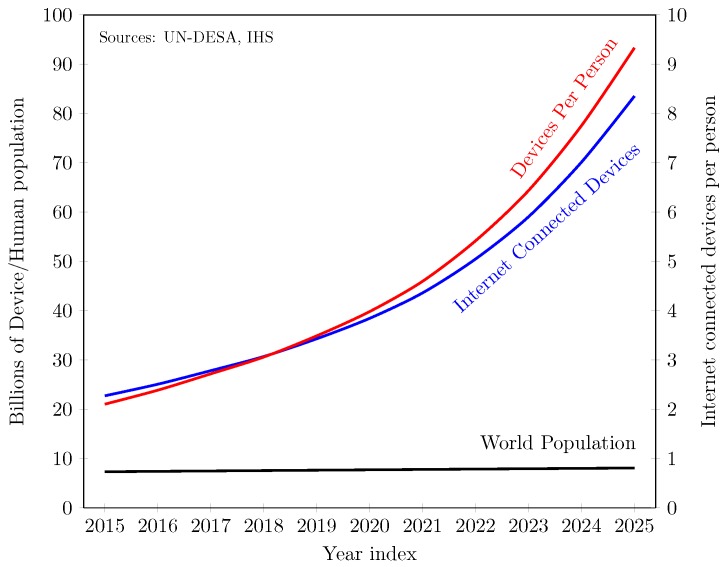
Internet-of-Things (IoT)-enabled devices connected to the Internet.

For the recent decades, Internet-of-Things (IoT) technology has been sharply propelling into our daily lives due to low-power and low-cost smart objects like sensors, controllers, actuators, etc. The widespread applications of IoT, including smart homes, smart meter, smart cities, eHelth, connected cars, and so on, require Internet connectivity for anything, anywhere, and anytime [1]. In one report, Cisco anticipates that the Internet-connected devices will be 2.7% of the worldwide physical objects by 2020, which is more than that of 80% in 2012 [2]. From another recent report [3], we have calculated based on the United Nation world population prospects [4], that each person will have at least nine devices on the Internet in 2025, as shown in Figure 1. On one hand, the leap in IoT growth can liberate humans from the old and dump devices, while it requires enabling protocols and more spectrum on the other. Worse still, the available spectrum is scarce; especially in the Industrial, Scientific, and Medical Bands (ISM). However, most of the IoT technologies (e.g., Bluetooth Low Energy, WiFi, 6LoWPAN, ZigBee, etc.) depend on ISM bands that are not really sufficient to their needs for spectrum.

Cognitive Radio (CR) is considered as a cornerstone technology to resolve the spectrum shortage issue through the dynamic spectrum allocation [5,6,7]. In CR networks, allocated frequencies are often used by the Primary Users (PUs), which have higher priority rights of spectrum use. Conversely, the Secondary Users (SUs) do not have a spectrum license, but they can dynamically occupy the unused frequencies (or white spaces) in the absence of PUs [8,9]. However, the CR system may not have the freedom to operate at the similar transmission parameters across the existing spectrum bands in order to avoid interference to the PUs [10]. Further, a CR system should accurately detect white spaces without generating a level of harmful interference, which is what is not acceptable to the PUs. Unfortunately, this is not always true because the accuracy of energy detector equipped with the CR system for spectrum sensing is not perfect due to inevitable inefficiencies like the false alarm and misdetection [11,12]. The spectrum assignment through dynamic access is, therefore, not trivial since the radio transmissions can interfere with each other unless separated enough in time, space or frequency. The dynamic spectrum access can be more aggressive as a channel can be used by multiple users simultaneously if only the channel satisfies a predefined quality condition. This situation will get worse if IoT-enabled SUs are densely deployed to act as competitors. Hence, the dynamic spectrum leasing, e.g., in [13,14], can increase the transmission power density and subsequently the interference level [15]. We need to consider the leaked powers from the adjacent SUs to the PU receivers especially in the decentralized networks due to hidden primary terminal problem [16,17,18].

In the existing literature, purpose-built Medium Access Control (MAC) protocols for CR-based IoT networks have not been studied directly. However, there exist several MAC protocols for CR networks as in [19,20,21,22,23], which always consider a dedicated Common Control Channel (CCC) to schedule the SUs’ transmissions in a sequential way. In [19], the authors proposed an opportunistic MAC protocol that relies on an exclusive CCC for each frame. In this protocol, the channel time is divided into two virtual segments named as report and negotiation phases. First, SUs report the sensing information of the available channels in the report phase. Then, in the negotiation phase, they compete to win the access opportunity for the next frame using a *p*-persistent CSMA/CA mechanism. To enable this protocol, each SU necessarily equipped with the two transceivers. That is, the one transceiver is tuned to the CCC, while the other is dedicated to sense and occupy the identified unused spectrum channels. In [22], the OMC-MAC is proposed, in which the CCC time is divided into non-overlapping periods called beacon intervals. Therein, each beacon interval consists of three phases called sensing, contention, and transmission phases. According to this protocol, SUs first sense channels in the sensing phase, then contend to win a transmission opportunity in the contention phase and they finally follow a contention-free process in the transmission phase. However, CCC-based MAC protocols are not much attractive due to denial of service attack, jamming, and single point of failure [24,25,26].

To the best of the authors’ knowledge, few of the previous works have considered the random access model that does not require an exclusive CCC and so it is more suitable for the CR-based IoT network. However, SUs’ channel access, and PUs’ detection and protection become more challenging in the absence of CCC. Furthermore, in distributed CR network the lack of centralized control without CCC can cause many challenges like the hidden primary terminal problem. We can find various MAC protocols for distributed CR network that based on the random access model, as in [27,28,29,30,31,32,33,34]. In [27], the VX, VAC, and KS schemes are proposed, which enable two metrics, to protect the transmission priority rights of the PUs, named collision probability and overlapping time in a random access mechanism. In these sachems, collisions between the SUs due to the hidden terminal problem are not considered, which are not trivial in the densely deployed secondary networks. Furthermore, the assumed scenario of one PU band and one SU is too simple, which is in fact an impractical assumption. In [29], the O-CSMA/CA is proposed, in which the backoff algorithm of the IEEE 802.11 standard is revised to improve fairness and reduce collisions among the SUs. In this protocol, SUs enable virtual carrier sensing to resolve the classical hidden and exposed terminal problems. However, no mechanism is suggested to resolve the hidden primary terminal problem to protect the PUs’ transmissions.

In [31,32,33], the authors suggested a two-level opportunistic spectrum access strategy in CR networks. In the first level, SUs enable spectrum sensing with a given probability to detect and protect the PUs. In the second level, they compete to access channel with one of the following random access mechanisms called CR-ALOHA and CR-CSMA protocols. The CR-ALOHA is based on the classical *Slotted* ALOHA scheme as that in [35], while the operation of CR-CSMA is inspired by the traditional carrier sense multiple access schemes as that in [36]. However, the performance of these protocols is not very attractive because of the inability to avoid packet collisions with nearby SUs. In [34], the CR-CSMA/CA is proposed to resolve the hidden primary terminal problem in distributed CR networks. In this scheme, the transmitter sends a control packet, called PTS, to initiate the mutual spectrum sensing with the receiver. When both the transmitter and the receiver confirm all the neighboring PUs to be silent, they can transmit a data packet following the RTS/CTS handshaking procedure as in the standard CSMA/CA [37]. However, this scheme is not much attractive because mutual spectrum sensing (a relatively long operation) is carried out before the RTS/CTS handshaking procedure that can lead to increase the cost of transmission failure. We notice that the event of transmission failure is most likely happen in a densely deployed network and so it should not have unnecessary overhead to maintain the efficiency of the system. In our previous works [38,39,40], we suggest different proposals of random access MAC protocols inspired by the CR-CSMA/CA.

In [38], the CR-MEGA is proposed, which adopts a dual sensing approach by enabling carrier sensing with spectrum sensing in a sequential way so as to avoid packet collisions with the nearby SUs and as well as the faraway PUs. This protocol performs well even with the highly active PUs due to its proactive mechanism. However, the advantage is achieved with the increased sensing overhead, which is what is not acceptable in a densely distributed environment. In [39], hybrid-MAC is proposed, which is based on the asynchronous and hybrid spectrum sensing techniques. This scheme alternatively exploits two different spectrum sensing approaches, i.e., fast sensing [41] and fine sensing [42], in addition to the traditional carrier sensing of CSMA/CA. In [40], the adaptive-MAC is proposed, which is the straightforward extension of the hybrid-MAC. In this scheme, a purpose-built algorithm in SUs controls the adaptive spectrum sensing time, depending on the packet transmission result and the previous activity of PUs, in order to maintain a balance between the system throughput and the spectrum sensing overhead. However, the performance of both hybrid MAC and adaptive-MAC are not very optimistic due to the same reason as that in CR-CSMA/CA protocol.

In [43], a random access model called Mutual Sense Multiple Access with Collision Avoidance (MSMA/CA) is proposed, which is based on cognitive radio network to efficiently utilize the bandwidth and spectrum resources in the IoT-enabled environment. MSMA/CA enables mutual spectrum sensing operation in the midst of the two control frames named Notify-To-Sense (NTS) and Acknowledge-To-Sense (ATS), to avoid the hidden primary terminal problem. The operation of MSAM/CA is rather smart and agile because it has merged the functionality of classical RTS/CTS into NTS/ATS frames with the added advantage of spectrum sensing. We notice that the long spectrum sensing period in the midst of NTS and ATS can let the hidden SUs affect the transmission in densely deployed networks. That is, with the NTS and ATS frames SUs transmitter and receiver can only stop the exposed and hidden SUs, respectively. However, if the gap between the sharing of NTS and ATS frames is large due to the long spectrum sensing period, then hidden SUs can also broadcast the NTS frame (followed by the secondary transmitter) and hence cause collision over the secondary receiver. We think that this phenomenon can severely affect the system performance when SUs are placed in close proximity in a super dense environment. It is, therefore, the optimum selection of the spectrum sensing period especially in the IoT environment is desirable. In addition to the spectrum sensing period, the performance of the energy detector is vulnerable by another factor, i.e., spectrum sensing threshold, due to the peculiarities of the energy detection sensing model.

In this paper, we design a novel MAC protocol for densely deployed CR-based IoT networks by modifying the prevalent CSMA/CA protocol. Our protocol is called Handshake Sense Multiple Access with Collision Avoidance (HSMA/CA) mechanism since it purposefully features the handshake based mutual spectrum sensing in the transmitter and the receiver. We tailored the Notify-To-Sense (NTS)/Clear-To-Sense (CTS)/Acknowledge-To-Sense (ATS) procedure for the data transmission in place of the NTS/ATS operation in existing MSMA/CA in order to improve the SUs’ access operation in a dense environment. The NTS/CTS/ATS mechanism first enables the handshaking operation among the SUs and then conduct the spectrum sensing operation in the transmitter and in the receiver to avoid the hidden secondary terminal and hidden primary terminal problems simultaneously. Moreover, we incorporate the optimization of the spectrum sensing period considering the inefficiencies of energy detector to improve the performance of the CR-based IoT networks.

The key contributions of this work are summarized as follows:

- We propose a new MAC protocol for densely deployed CR-based IoT networks that resolves the hidden primary terminal problem with the minimal possible overhead while effectively dealing with the classical hidden (secondary) terminal problem.
- We develop an optimization model to judicially adapt the spectrum sensing period considering the incidences of false alarm and misdetection and hidden primary terminal, in order to improve the system efficiency and reduce the interference to active PUs.
- We analyze the performance of our proposed protocol in terms of normalized throughput with the Markov chain model and compare the results with that of existing CR-MEGA and MSMA/CA protocols.

The rest of this paper is structured as follows. Section 2 covers the main issues in CR-based IoT networks that make them different from the classical wireless networks. Section 3 takes an overview of the system model. Section 4 describes the proposed MAC protocol. Section 5 analyzes the proposed protocol through a mathematical model. Section 6 validates our model and discusses the obtained results. In the last section, we summarize and conclude the paper.

## 2. Issues of CR-Based IoT Networks

There are a variety of reasons that can compromise the performance of the classical wireless networks. However, we here only discuss the issues related to the CR-based IoT networks as in the following.

### 2.1. Heterogeneous Environment

A CR system generally works in a multi-dimensional environment having coexistence of PUs and SUs with variations of time, space and frequency. The coexistence of non-cooperative users makes it very hard to sense the signals from PUs reliably. Moreover, the resulted interference induced from autonomous detection procedure of multiple SUs in an independent sensing environment would also lead to confuse the CR system by overestimation of signal’s power. Hence, the potential transmitter is likely to deceive by assuming the inactive status of PU to be active. This phenomenon is more likely to happen in dense networks.

### 2.2. Tradeoff between Sensing Schemes

Spectrum sensing is to detect the signal from PUs possibly under noisy environment. It is very crucial to identify the PU’s signal for the communication of SUs at the desired interference level. There are two general techniques on spectrum sensing i.e., fast sensing and fine sensing [44]. The fast sensing measures the energy level of received signals while consuming less than 1 ms. This approach has the advantage of reporting results in a very short time, but it cannot distinguish the source of signals. On the other hand, the fine sensing consumes several tens of milliseconds, and identifies better the signal source by using the matched filter or cyclostationary detection. Hence, there exists a tradeoff between sensing time and sensing quality alternatively in addition to the selection of an appropriate sensing scheme.

### 2.3. Sensing Performance

The perfect sensing is a big challenge in real environments. There exists always a probability of misdetection and false alarm. We denote the false alarm probability as α and the misdetection probability as β, respectively. The false alarm means that an SU mistakenly detects an idle PU to be active. Then the SUs can miss the transmission opportunity. For SU *i*, the false alarm probability, $\alpha_{i}$, can be found in [45] as,

(1) $$\alpha_{i}=Q\left(\left(\frac{\lambda }{\nu^{2}}-1\right)\sqrt{Tf_{s}}\right),$$

where λ is the detection (or spectrum sensing) threshold of the sensor to decide the activity of PU, and $\nu^{2}$ denotes the power of the noise in the channel. On the other hand, misdetection indicates that an SU recognizes active PUs as idle by mistake, which leads to the significant interference due to subsequent transmissions of SUs. In [45], the detection probability of an arbitrary SU *i* is derived as,

(2) $$\Omega_{i}=Q\left(\frac{1}{\sqrt{2\gamma +1}}Q^{-1}\left(\alpha_{i}\right)-\sqrt{Tf_{s}}\gamma \right),$$

where *T* denotes the spectrum sensing time, $f_{s}$ indicates the sampling rate of the channel, γ accounts for the Signal-to-INoise-Ratio (SINR) of the PU’s signal measured at the SU *i*, and Q(·) represents the complementary distribution function of a standard Gaussian variable. Hence, the misdetection probability, $\beta_{i}$, of an SU *i* can be written as,

(3) $$\beta_{i}=1-\Omega_{i}.$$

In order to adequately protect the PUs, we require the misdetection probability should be bounded by a predefined value $\hat{\beta }$ such that $\beta \leq \hat{\beta }$.

### 2.4. Hidden Primary Terminal Problem

The performance of an CR system is significantly affected by its sensing ability during the detection process of the active PUs. SUs may fail to detect the presence of PUs due to multipath fading affects in wireless channels and temporal and spatial appearance of the PUs. In that case, SUs can cause much interference to the PUs in their successive transmissions.

**Figure 2:**
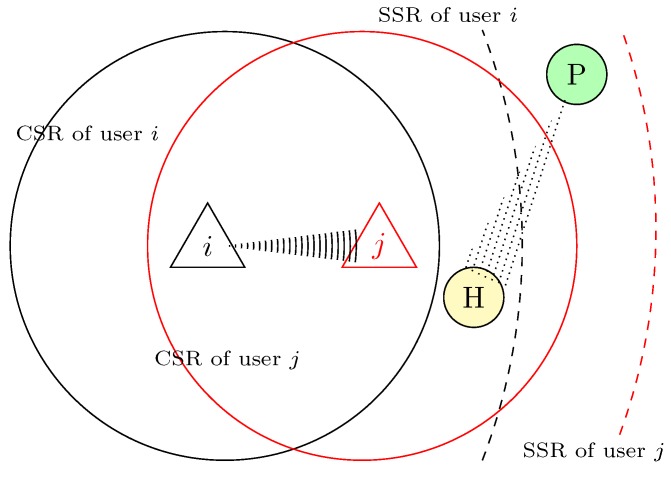
Example scenario of the hidden primary terminal problem. CSR, Carrier Sensing Range; SSR, Spectrum Sensing Range; P, Primary Terminal; H, Hidden Primary Terminal.

We illustrate the scenario of the hidden primary terminal problem in Figure 2, where the solid lines denote the Carrier Sensing Range (CSR) and the dotted lines indicate the Spectrum Sensing Range (SSR), respectively. We see that SUs *i* and *j* are respectively involved in a transmission as a transmitter and as a receiver. We can also observe that PUs *P* and *H* are respectively located outside the CSRs of the SUs *i* and *j*. In carrier sensing, SU *i* can only ensure the inactive status of PUs that are located inside its CSR, when it senses the channel is idle for Data Interframe Space (DIFS) interval, but not that of PU *H*. In spectrum sensing, the ability of SU *i* is, however, only limited to evaluate when PU *H* is working as a transmitter but not when it is performing as a receiver. The obvious reason is the inability of SU *i* to receive signals of the PU *P*’s transmission to the hidden PU *H*. Hence, SU *i*’s transmission to SU *j* can essentially affect the likely transmission of PU *P* to the PU *H*. In such a scenario, a secondary network can create harmful interference when an PU e.g., *H*, is hidden from SU e.g., *i*. It is very desirable that SU *i* should make sure of the silence of neighboring PUs when it involves in transmission with SU *j*.

## 3. System Model

We have considered one secondary network with *K* SUs and one primary network with multiple PUs in the system. The SUs and PUs both can coexist yet cannot communicate with each other since they lack collaboration. The secondary network is established in a decentralized manner without getting support from the centerlized server, in which SUs’ transmissions are carried out through peer-to-peer communication setup. However, SUs, whenever they act as IoT devices, can rely on the server for Internet service. We have assumed the single and shared channel model, therein SUs can find transmission opportunities whenever the legitimate owners (or incumbent PUs) are inactive. Once PUs are active, SUs are then liable to vacate the channel immediately. To identify a transmission opportunity, the secondary transmitter and its corresponding receiver, denoted as SU *i* and SU *j*, can enable spectrum sensing using one of the energy detection, autocorrelation-Based Sensing or cyclostationary feature detection mechanisms [45,46,47,48].

During a spectrum sensing operation, each SU in its neighborhood can detect PUs either to be active or inactive. The PUs in the neighborhood of SU *i* can be found active with the probability of $\pi_{1,i}$, while the neighboring PUs can be observed as inactive with the probability of $\pi_{0,i}$. However, the spectrum sensing operation is not ideal due to the imperfect sensing environment. We hence consider the incidence of false alarm and misdetection in order to make the proposed system more realistic. Ultimately, SU *i* can conduct a data transmission operation based on the sensing results, provided that the channel is clear from the incumbent PUs. For SU *i*, the clear-channel probability (*R*), with which the channel is free from the incumbent PUs, can be obtained as,

(4) $$R_{i}=\left(1-\alpha_{i}\right)\pi_{0,i}+\beta_{i}\pi_{1,i},$$

where recall that $\alpha_{i}$ and $\beta_{i}$, respectively refer to the probabilities of false alarm and misdetection for SU *i*, as defined in Equations (1) and (3). So, the energy detector in SU *i* suggests declaring the activity or inactivity of incumbent PUs with probability $1-R_{i}$ and probability $R_{i}$, respectively.

## 4. Proposed MAC Protocol

We here discuss the proposed MAC protocol called Handshake Sense Multiple Access with Collision Avoidance (HSMA/CA), which is designed for the densely deployed cognitive radio-based IoT networks. In HSMA/CA, the system time is divided into consecutive and multiple equal sized time slots, therein SUs competing at a moment follow a random access procedure over the shared channel. The channel is based on a single channel model, in which data and control packets both are transmitted over the same channel. SUs contend at the beginning of each time slot, denoted as σ, and the winner SU thereafter transmits data packet according to a defined mechanism outlined in the following.

### 4.1. NTS/CTS/ATS Access Mechanism

Our proposed protocol, HSMA/CA, is a tailor-made version of the standard CSMA/CA (and MSMA/CA) for the densely distributed systems. In classical CSMA/CA, each station must first identify the state of the channel as idle or busy through the carrier sense before it enables collision avoidance and data transmission procedures. Therein, carrier sensing includes sensing of the physical medium by Clear Channel Assessment function to avoid a transmission overlap and the Network Allocation Vector (NAV) function to reserve the channel for data packet transmission. However, carrier sensing in CSMA/CA is limited because it is usually carried out at high SNR regime, and so vulnerable to the presence of hidden primary terminal nodes in CR ad hoc networks. For example, if an PU is positioned outside the carrier sensing range of an SU transmitter, it is hard to identify the activity status of this PU through the carrier sensing mechanism. On the other hand, the spectrum sensing method such as energy detection, autocorrelation-based sensing or cyclostationary feature detection mechanisms [45,46,47,48] can be carried out in low SNR regime, but requires significant time while introducing greater incidences of misdetection and false alarm. In HSMA/CA, we have used Notify-to-Sense (NTS)/ Clear-to-Sense (CTS)/Acknowledge-to-Sense (ATS) based handshake access mechanism in place of the classical RTS/CTS. The NTS/CTS/ATS procedure is purposefully designed to complement carrier sensing with spectrum sensing in order to avoid and protect the hidden secondary and hidden primary terminals, respectively. However, SUs under HSMA/CA adopt the similar backoff procedure as that is used in CSMA/CA since both protocols are based on the same random access model.

**Figure 3:**
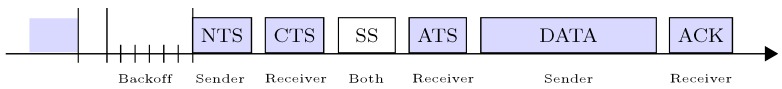
Typical packet transmission procedure under Handshake Sense Multiple Access with Collision Avoidance (HSMA/CA). NTS, Notify to Sense; CTS, Clear to Sense; SS, Spectrum Sensing; ATS, Acknowledge to Sense; ACK, Acknowledgment.

We illustrate the packet transmission procedure in accordance to HSMA/CA in Figure 3, which is described as follow. The SU with packet to send first chooses a backoff counter at random to avoid collision. Then the SU conducts the carrier sensing for one DIFS interval to check whether the channel is free or not and to avoid a transmission overlap. If the channel is sensed as idle for the DIFS interval, then SU decreases its backoff counter one by one for each backoff slot time observed as idle. In Figure 3, the chosen backoff counter is 6. If no transmission by other SUs is observed until the backoff counter becomes 0, the transmitter broadcasts an NTS packet to synchronize the spectrum sensing operation with the corresponding receiver. The NTS packet keeps the neighboring SUs silent by updating their NAVs until the spectrum sensing is done. If the receiver overhears the NTS packet, it will then send an CTS packet to the transmitter. The CTS packet makes the hidden SUs silent by updating their NAVs until the packet transmission is over. Once the NTS and CTS packets are exchanged successfully, the transmitter and receiver both can mutually conduct the spectrum sensing operation to protect the PUs in their neighborhood. After the mutual septum sensing, if there is no hidden PU active around the receiver, it returns the ATS packet to the transmitter. If the hidden PU is active otherwise, the receiver will be blocked by spectrum sensing, and so it will hold the ATS packet. Meanwhile, the transmitter will wait for the ATS packet assuming it is not blocked by the active PUs in its neighborhood. The transmitter sends the DATA packet when it decodes the ATS packet successfully, and the receiver returns the ACK packet at the end. If there is an error-free environment the transmitter decodes the ACK packet correctly and completes one DATA packet transmission.

We mention that an PU can receive interference when it becomes active during the exchange of NTS and CTS. However, such interference is acceptable in CR systems because of the following two reasons. First, IEEE 802.22 standard requires that SUs should necessarily vacate the channel within 100 ms once PUs become active [49]. For example, assuming the transmission rate of 1 Mbps and the packet size of 1000 bytes, the packet transmission time is 8 ms, much less than “100 ms”. Hence, the allowed time is good enough for the exchange of NTS and CTS packets. Second, the activity rate of incumbent PUs for cognitive radio networks is usually observed very low [50,51,52]. Hence, incumbent PUs are not likely to remain active most of the time. Refer to the exchange of NTS and CTS packets, it should be noticed that transmitter does not always receives the CTS packet when there is a collision among multiple NTS packets. The detailed operation under HSMA/CA is studied in Section 5.

### 4.2. Spectrum Sensing Optimization

We now provide an analysis to demonstrate how the performance of the proposed protocol could be improved under the real environments while considering the inefficiencies of spectrum sensing. We optimize the spectrum sensing period and the sensing threshold because both of the parameters can otherwise affect the performance of the proposed protocol. To improve the system performance, we can optimize both of the parameters by minimizing the incidence of false alarm and misdetection with a given activity rate of hidden primary terminals (or hidden PUs). In our model, we assume *N* spectrum sensing slots in a spectrum sensing period. We denote the duration of each sensing slot as Ts. Hence, the length of a spectrum sensing interval can be written as,

(5) T=N×Ts,

which is our design parameter. From Equations (1) and (3), we can derive false alarm and misdetection probabilities at a given sensing interval, so as to calculate the network throughput. Let the hidden PUs are activated with an activity factor *A* at each sensing slot time. Let *h* be the number of hidden PUs. We can find the hidden PUs’ interference probability, $P_{i}$, as a function of *N*, *A*, and *h* as,

(6) $$P_{i}=1-{\left[{\left(1-A\right)}^{h}\right]}^{N},$$

where the second term refers to the no-interference probability to the hidden PUs. We hence can derive the throughput of the system as,

(7) $$\tilde{S}=R\left(\lambda ,N\right)\times \left(1-P_{i}\right)C,$$

where *C* is a given channel capacity and recall that *R* is the clear channel probability as defined in Equation (4). From Equation (1), we can see that,

(8) $$\lambda =\hat{\lambda }\times \nu^{2},$$

where $\hat{\lambda }$ is the normalized value of the sensing threshold, λ and noise power, $\nu^{2}$ in the wireless channel. Please note that the optimization model is only designed to obtain the optimized value of sensing interval while considering the peculiarities of spectrum sensing in a real environment. So, the network throughput is derived to observe the effects of our design parameters. We have investigated the performance of our proposed protocol with more detail in the following.

## 5. Performance Analysis

We here demonstrate the performance of proposed MAC in terms of normalized throughput. Before we proceed, we have summarized the used symbols in Table 1.

### 5.1. Packet Transmission Process

**Figure 4:**
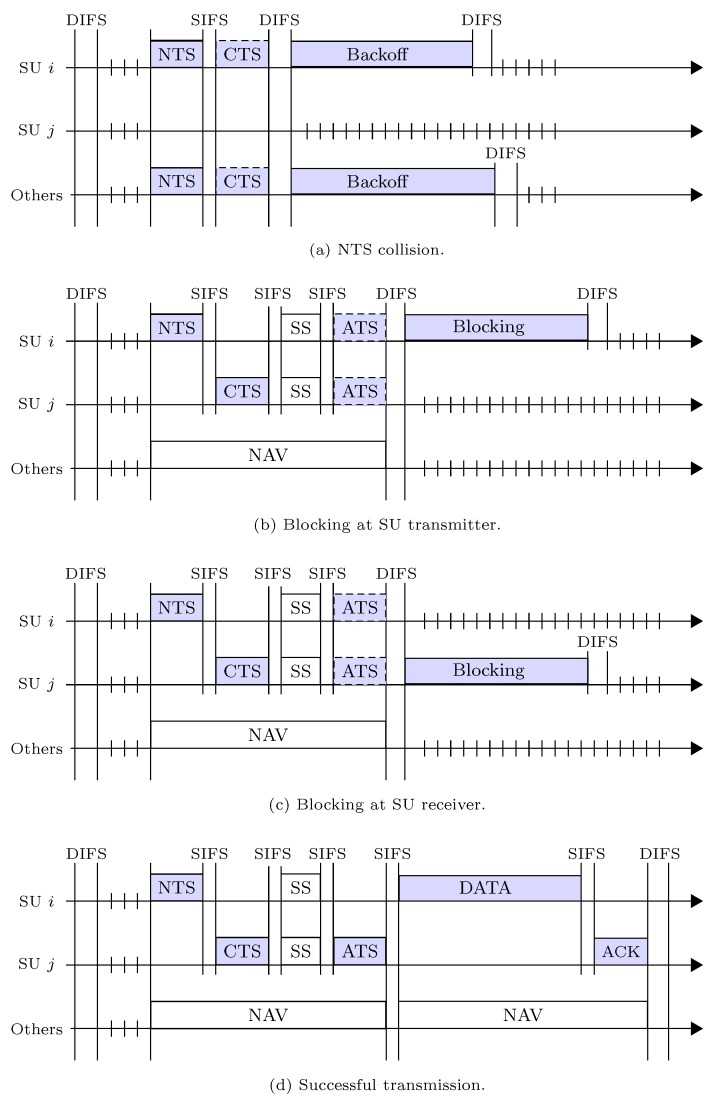
HSMA/CA access mechanism followed by the all possible events.

We investigate the packet transmission process of our HSMA/CA protocol. In this process, there exists one of the four possible events during a packet transmission process, which we called as: (1) NTS collision; (2) Blocking at SU transmitter; (3) Blocking at SU receiver; and (4) Successful transmission, as shown in Figure 4. These four events in one packet transmission attempt of SU *i* are denoted as $e_{i},i=1,\cdots ,4$. We calculate the probability (*p*) and time delay (*t*) of each event in the following.

#### 5.1.1. NTS collision

We recall that under HSMA/CA the contending SUs are dispersed with the backoff mechanism to avoid the collision. The backoff time of each SU is defined by its randomly chosen backoff counter, which is decreased by one whenever the backoff slot is idle. The winner SU whose backoff counter expires first can transmit the NTS packet. However, more than one winner SUs can cause NTS collision whenever their backoff counters expire at the same time. Suppose that SU *i* has the backlogged buffer queue and the backoff counter 0. So, SU *i* transmits NTS packet to the SU *j* with the transmission trial probability denoted as $X_{i}$. After the timeout, SU *i* is failed to receive the CTS packet since its NTS packet becomes collided with another NTS packet. That situation, what we called as event $e_{1}$, will happen with the probability,

(9) $$p_{1}=R_{i}\left(1-\prod_{k=2}^{K}\left[1-X_{k}R_{k}\right]\right),$$

where $X_{k}$ and $R_{k}$ respectively refer to the packet transmission trial probability and the clear channel probability for SU *k*. In the second term of Equation (9), this can be helpful to notice that NTS packet of at least one SU will collide to the NTS packet of SU *i* when *k* SUs, where k=2,…,K, are not trying to transmit with $1-X_{k}$ probability. From Figure 4a, time delay of SU *i* due to event $e_{1}$ is,

(10) $$t_{1}=\mathrm{NTS}+\mathrm{CTS}+\mathrm{SIFS}+\mathrm{DIFS},$$

where NTS and CTS accounts for the transmission time of one RTS and that of one CTS, respectively.

#### 5.1.2. Blocking at SU Transmitter

Suppose that SUs *i* and *j* have exchanged the NTS and CTS packets. Therefore, both SUs, thanks to NAV mechanism, mutually conduct their spectrum sensing operations without receiving interruption from the other SUs. However, SU *i* has sensed the neighboring PUs active and so it is blocked by the spectrum sensing for a predefined period, which is event $e_{2}$ that encounters with probability,

(11) $$p_{2}=1-R_{i}.$$

From Figure 4b, time delay by the event $e_{2}$ at SU *i* can be written as,

(12) $$t_{2}=\mathrm{NTS}+\mathrm{CTS}+\mathrm{SS}+3\mathrm{SIFS}+\mathrm{DIFS},$$

where SS denotes the length of spectrum sensing time for one mutual operation.

#### 5.1.3. Blocking at SU Receiver

Suppose that SU *i* is not blocked by the spectrum sensing, so it is waiting to receive the ATS packet from the SU *j*. However, if SU *j* has sensed the hidden PUs as active, then it will be blocked by the spectrum sensing operation and so cannot send the ATS packet. That is event $e_{3}$, in which SU *i* transmits packet to SU *j* with the packet transmission probability $\theta_{ij}$. However, the timeout in SU *i* will ultimately fail the data packet transmission with probability,

(13) $$p_{3}=R_{i}\left(1-R_{j}\right)\sum_{j\neq i}\theta_{ij}\left(\prod_{k=3}^{K}\left[1-X_{k}R_{k}\right]\right),$$

where $1-R_{j}$ denotes no channel clearance probability for SU *j*. The last term in Equation (13) indicates that no transmitter out of *k* SUs, where k=3,…,K, can interrupt SU *i*’s transmission. From Figure 4c, the time delay due to event $e_{3}$ can be measured as,

(14) $$t_{3}=\mathrm{NTS}+\mathrm{CTS}+\mathrm{SS}+3\mathrm{SIFS}+\mathrm{DIFS}.$$

#### 5.1.4. Successful Transmission

Now we consider the successful transmission case, which we called as event $e_{4}$. Suppose that SUs *i* and *j* are not being blocked by the mutual spectrum sensing operation. In that case, SU *i* will positively decode the ATS packet if there is an error-free channel. Thereafter, SU *i* sends an DATA packet and then it will correctly receive the ACK packet from the corresponding SU *j*. Meanwhile, NAV mechanism keeps the other SUs silent for the respective time periods noticed in the overheard NTS, CTS, and ATS packets. The occurrence probability of event $e_{4}$ can be written as,

(15) $$p_{4}=R_{i}R_{j}\left(1-X_{j}\right)\sum_{j\neq i}\theta_{ij}\left(\prod_{k=3}^{K}\left[1-X_{k}R_{k}\right]\right),$$

where $1-X_{j}$ represents reception state of SU *j*. From Figure 4d, time delay by event $e_{4}$ is given as,

(16) $$\begin{array}{cc} t_{4} & =\mathrm{NTS}+\mathrm{CTS}+\mathrm{SS}+\mathrm{ATS}+\mathrm{DATA}\\ & \;\;\;\;\;\;\;\;\;\;\;\;\;\;\;\;\;\;\;\;\;\;\;\;\;\;\;\;\;\;\;\;\;+\;\mathrm{ACK}+5\mathrm{SIFS}+\mathrm{DIFS}, \end{array}$$

where ATS, DATA, and ACK respectively denote the transmission time of one ATS packet, transmission time of one DATA packet, and that of one ACK packet.

### 5.2. Normalized Throughput

We analyze the performance of our proposed HSMA/CA protocol in terms of normalized throughput, which is what is derived under the given set of assumptions:

- The topology of the secondary network is composed of a fully connected complete graph, therein SUs are directly connected to each other with a single hop distance.
- The secondary network is saturated such that SUs have non-empty queues, in which there is always an DATA packet to send at each station.
- The transmission channel is error-free and there is no capture effect, so the packets are discarded when they receive collision.
- The control and DATA packets are sent through a single channel, which is shared among the SUs.
- The SUs use the same physical layer and their data transmission rate is also constant.

**Figure 5:**
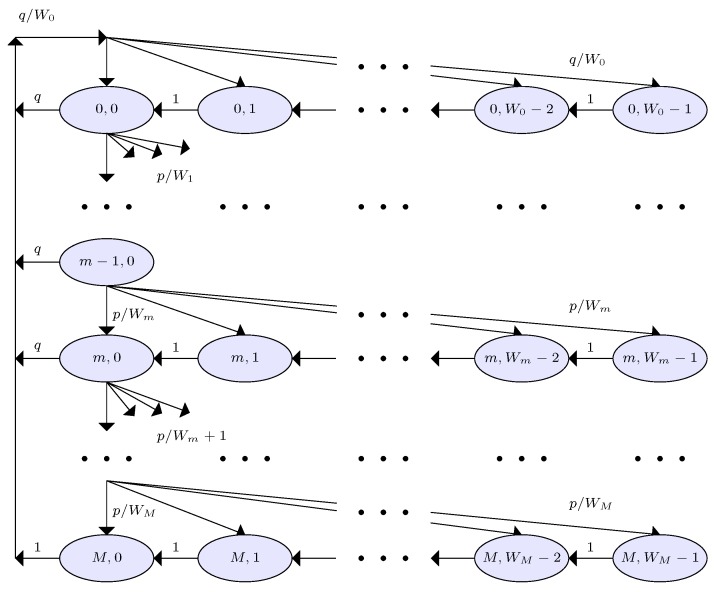
Markov chain model of the backoff process under HSMA/CA system. *p*, Failed transmission probability; *q*, Successful transmission probability; *m*, Backoff counter; *M*, Maximum retry limit; $W_{0}$, Initial window size; $W_{M}$, Maximum window size.

We investigate the backoff process of SUs in HSMA/CA system with a two-dimensional Markov chain model as illustrated in Figure 5, in which backoff states at time *t* are defined by the values of two random processes of backoff stages m(t) and backoff counters c(t). We can denote the backoff state of an arbitrary SU *i* being the value of a backoff stage m−1 and that of a backoff counter c−1 as (m,c). The arbitrary SU *i* decreases it backoff counter from its contention window *W* whenever the channel is observed as idle. Once the backoff counter reaches 0, SU *i* can begin data transmission process. In that process, if the transmission is successful, then SU *i* returns to its initial backoff stage. Conversely, when SU *i* observes collision, it increases its backoff stage from *m* to m+1 upto a maximum retry limit *M*. In that case, the backoff counter is chosen among $m\in [0,W_{m+1}]$ provided m≤M. We denote the size of initial and that of maximum window as $W_{0}$ and $W_{M}$, respectively. When SU *i* transmits with probability *p*, it is a failed transmission that happens either due to $e_{1}$, $e_{2}$, or $e_{3}$. For SU *i*, we can obtain the probability of a failed transmission from Equations (1), (9), (11) and (13),

(17) $$p_{i}=\alpha_{i}+p_{1}+p_{2}+p_{3},$$

which will be increased by the inevitable probability of false alarm, $\alpha_{i}$, in a real environment. From Equations (3) and (15), we can obtain the probability of a successful transmission as,

(18) $$q_{i}=\beta_{i}+p_{4},$$

where $p_{4}$ remains high due to misdetection probability, $\beta_{i}$, in a real sensing environment. From Figure 5, we can obtain the state transition probabilities of the Markov chain as Equation (20). We noticed that the Markov chain of our HSMA/CA protocol hase close resmblace to that in Chong et al. [53] due to the common backoff process. We hence directly refer to the results in Chong et al. [53] for the transmission trial probability of SU *i* as Equation (21).

We can distinguish system slots into empty slots and event slots. In the empty slots, SUs do not attempt to transmit packets. However, SUs in the event slots observe one of the four possible events, as defined in Equations (10), (12), (14) and (16), whenever they attempt to transmit data packets. We can readily obtain the occurrence probability of the empty slots as,

(19) $$P_{e}=\prod_{i}\left(1-X_{i}\right).$$

(20) $$\left\{\begin{array}{cc} P\left\{\left(0,c\right)\to \left(m,0\right)\right\}=\frac{q}{W_{0}-1}\; & \mathrm{for}\;m\in \left[0,M-1\right],\;c\in \left[1,W_{m}-1\right)\\ P\left\{\left(m,c\right)\to \left(m-1,0\right)\right\}=\frac{p}{W_{m}-1}\;\; & \mathrm{for}\;m\in \left[1,M\right],\;c\in \left[1,W_{m}-1\right)\\ P\left\{\left(0,c\right)\to \left(M,0\right)\right\}=\frac{1}{W_{M}-1}\;\; & \mathrm{for}\;c\in [1,W_{m}-1), \end{array}\right.$$

(21) $$X_{i}=\sum_{m=0}^{M}\left(\frac{2(1-2p)(1-p)}{W\left(1-{\left(2p\right)}^{M+1}\right)\left(1-p\right)+2\left(1-2p\right)\left(1-p^{M+1}\right)}\right).$$

We can further classify the event slots into transmission slots and no-transmission slots. The slots in which transmissions happen successfully are transmission slots. From Equation (18), we already have obtained the successful transmission probability under a real environment for SU *i*. Hence, the occurrence probability of the slots with successful data transmission can be written as,

(22) $$P_{r}=\sum_{i}X_{i}q_{i}.$$

However, the no-transmission slots are those slots who have observed the failed transmissions. We can find the occurrence of no-transmission slots with probability $P_{n}=1-\left(P_{e}+P_{r}\right)$.

The throughput of the proposed HSMA/CA system can be expressed as,

(23) $$S=\frac{\mathrm{Average}\;\mathrm{data}\;\mathrm{paylod}\;\mathrm{bits}\;\mathrm{transmitted}\;\mathrm{in}\;a\;\mathrm{slot}}{\mathrm{Average}\;\mathrm{length}\;\mathrm{of}\;a\;\mathrm{slot}}.$$

We assume an autonomous system in which SUs have an average size of the data payload bits E[P]. So, the average amount of the data payload bits successfully transmitted in one slot is $P_{t}E\left[P\right]$. Let E[O] be the average length of a slot, which can intuitively be computed as,

(24) $$E\left[O\right]=P_{e}\sigma +P_{r}E\left[T_{r}\right]+P_{n}E\left[T_{n}\right],$$

where $T_{r}=t_{4}$ and $T_{n}=t_{2}\left(=t_{3}\right)$. We assume $t_{1}\left(=t_{2}=t_{3}\right)$ so that all SUs can keep pace in the system. Hence, $T_{n}=t_{1}\left(=t_{2}=t_{3}\right)$. From Equation (16), we can observe that

(25) $$\begin{array}{cc} E[T_{r}] & =\mathrm{NTS}+\mathrm{CTS}+\mathrm{SS}+\mathrm{ATS}+\mathrm{DATA}\\ & \;\;\;\;\;\;\;\;\;\;\;\;\;\;\;\;\;\;\;\;\;\;\;\;\;\;\;\;\;\;\;+\;\mathrm{ACK}+5\mathrm{SIFS}+\mathrm{DIFS}\\ & =\mathrm{NTS}+\mathrm{CTS}+\mathrm{SS}+\mathrm{ATS}(H+E[P])/E\\ & \;\;\;\;\;\;\;\;\;\;\;\;\;\;\;\;\;\;\;\;\;\;\;\;\;\;\;\;\;\;\;+\;\mathrm{ACK}+5\mathrm{SIFS}+\mathrm{DIFS}, \end{array}$$

where *H* and *E* respectively represent the size of PHY plus MAC headers and the channel transmission rate. From Equation (12), $t_{2}$ is constant and so $E\left[T_{n}\right]=t_{2}$. Conclusively, Equation (23) becomes,

(26) $$S=\frac{K-1\left(P_{t}E\left[P\right]\right)}{K\left(P_{e}\sigma +P_{r}E\left[T_{r}\right]+P_{n}E\left[T_{n}\right]\right)},$$

where the term $\frac{K-1}{K}$ refers to the blocking effect such that only one SU can at most be blocked due to spectrum sensing out of *K* users in the system.

## 6. Results and Discussion

We used energy detection model for spectrum sensing, in which first we need to determine the optimized value of the normalized threshold and that of spectrum sensing slots. For that purpose, we have derived the spectrum sensor’s false alarm and misdetection probabilities as Equations (1) and (3), respectively. We assume a 1 MHz channel and set the spectrum sensing sampling rate with 6 MHz and SNR of the PU signal measured at SU is set to 0 dB. Further, the number of spectrum sensing slots is assumed to be 20, with duration of 0.035 ms each. We consider the activity rate of hidden PU as 1%. To validate our analysis, we develop simulation in C++ code according to the details of our HSMA/CA protocol. We average 1000 runs to obtain one simulation result. In a nutshell, the default parameters used in the simulation are summarized in Table 2.

### Simulation Results

**Figure 6:**
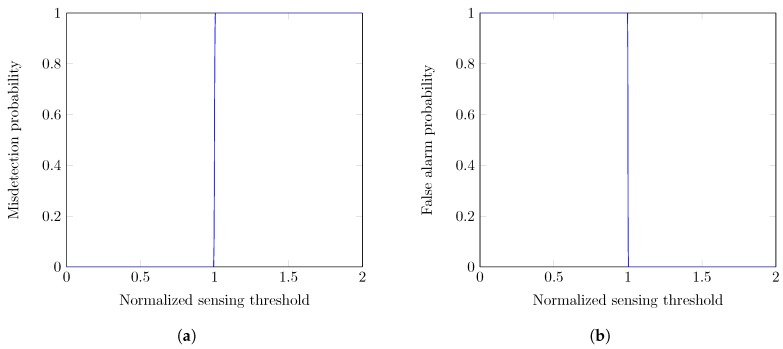
Optimization of normalized sensing threshold (at SNR=0). (**a**) Misdetection probability vs. normalized sensing threshold; (**b**) False alarm probability vs. normalized sensing threshold.

In Figure 6, we have shown the effect of various design parameters over the spectrum sensing errors in the system. We have evaluated the variations of misdetection probability for the various values of normalized sensing threshold in Figure 6a. We can see that misdetection probability remains 0 when the threshold is less than 0.9975, and it becomes sharply increases when the threshold is greater than 0.9975. We mention that the sensing threshold is bounded upper due to the requirement on the misdetection probability as ≤0.1 (or detection probability as ≥0.9). With higher sensing threshold (i.e., >0.9975), the throughput of secondary network increases but the PUs cannot protect enough. On the other hand, the false alarm probability remains high with threshold less than 0.9975 and then it goes down sharply when the threshold exceeds 0.9975, as shown in Figure 6b. Hence, the achieved optimized spectrum sensing threshold by minimizing the spectrum sensing errors is 0.9975.

**Figure 7:**
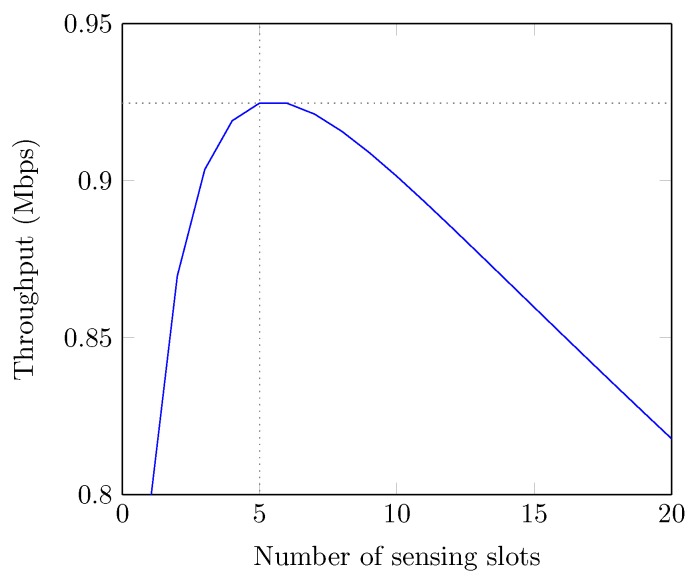
Number of sensing slots optimization.

**Figure 8:**
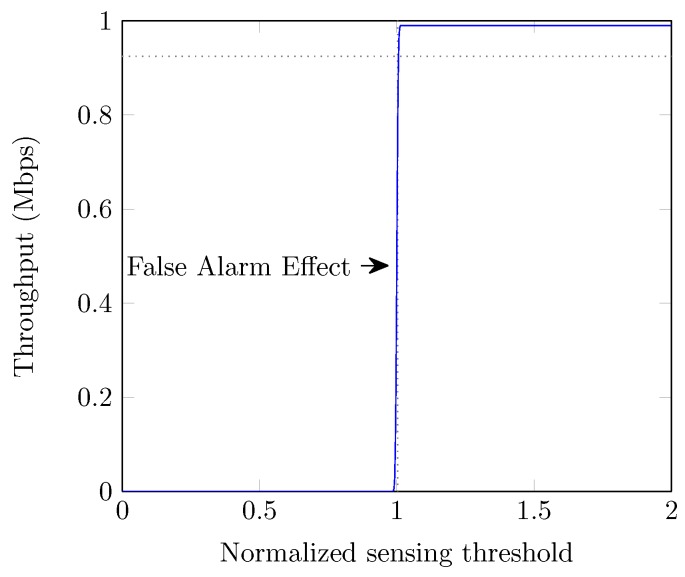
Throughput vs. normalized sensing threshold.

Figure 7 illustrates the effect of the number of spectrum sensing slots over the system throughput. We observe that the achieved throughput of the system increases with the increase in the number of sensing slots until a certain limit and then decreases monotonically. With less number of sensing slots, channel resources are wasted due to the high probability of false alarm and thus the system performance degrades. Conversely, with a large number of sensing slots, the system throughput can decrease due to the increased sensing overhead. We can also see that there exists the optimal number of sensing slots (=5) and accordingly the optimal spectrum sensing interval (=0.035 ms × 5) for the best throughput. However, it depends on the system environment such as channel model, sampling rate and the distance between PUs and SUs. In Figure 8, we exhibit the effect of sensing threshold over the throughput of the system. We can see that system throughput remains low (=0) below the optimal threshold and thereafter it increases up to the maximum value due to the higher probability of false alarm and misdetection, respectively. If the chosen threshold is higher than the optimal value, the throughput of the system can be achieved further but at the cost of higher misdetection, which is not desirable due to the protection priority of PU in CR system.

**Figure 9:**
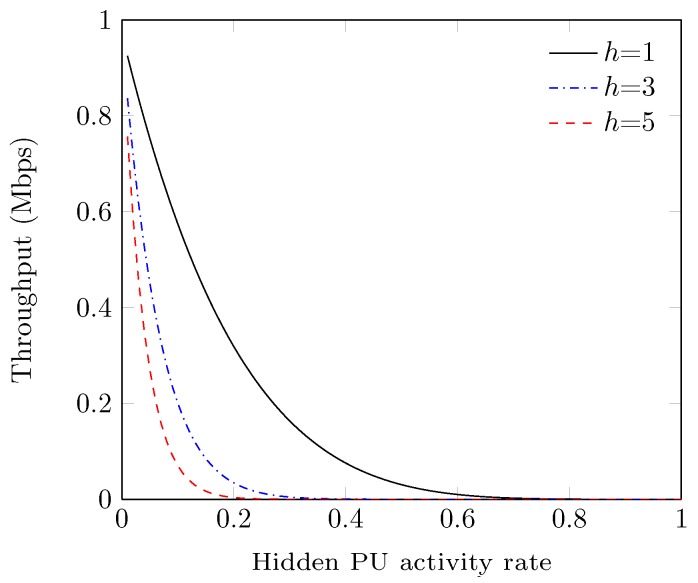
Throughput vs. hidden PU activity rate.

We have shown the effect of hidden primary node activity rate over the system performance for the various number of hidden PUs in Figure 9. Therein, the values of throughput monotonically decrease with the increase in activity rate of the hidden primary node. This is because of the fact that a hidden PU can interrupt more with higher activity rate and secondary network accordingly vacates the channel to avoid interference that eventually leads to a decrease in system performance. However, the gap between the curves of throughput is attributed due to the large number of hidden PUs, that can block SUs with more probability and so the achieved throughput of the system decreases sharply.

**Figure 10:**
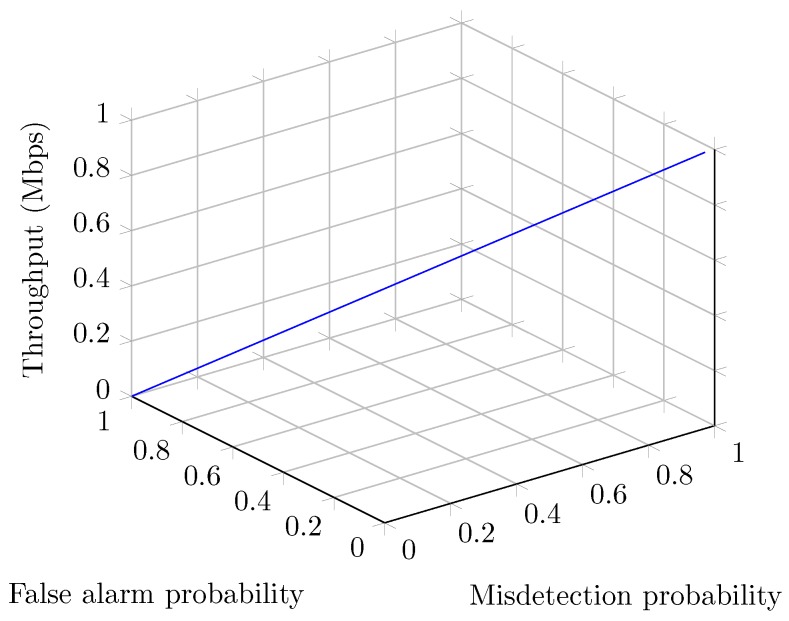
Throughput vs. sensing error probabilities.

Figure 10 illustrates the effect of sensing errors in terms of false alarm and misdetection probabilities at the optimal values of spectrum sensing slots and normalized threshold. We can see that as long as the false alarm probability decreases and the misdetection probability increases, the throughput of the system increases accordingly. This is because of the fact that a smaller value of false alarm and a larger value of misdetection can generate the higher value of throughput. On the contrary, if the sensing errors and spectrum sensing slots in the system are not optimized, then SUs’ system performance and PUs’ protection both can degrade significantly.

**Figure 11:**
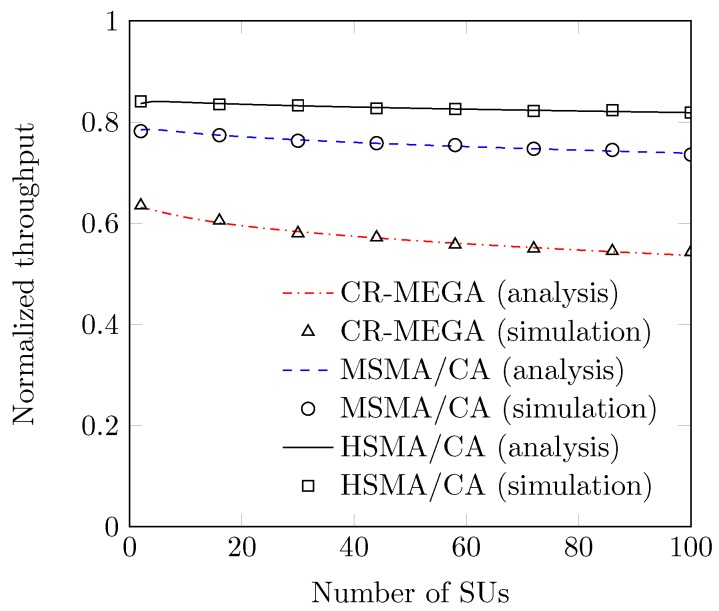
Normalized throughput vs. number of SUs.

We now present the performance of proposed protocol in terms of normalized throughput. In Figure 11, we compare the performance of the proposed HSMA/CA with CR-MEGA and MSMA/CA protocols. We witness that our analysis is very accurate because our analysis results closely follow the simulation results. We observe that the performance of the secondary network remains low since the contention window becomes large for the less number of SUs. On the other hand, contention window becomes smaller for a large number of SUs that lead to increase the RTS collisions. So, the system performance is decreased. We also observe that our proposed HSMA/CA outperforms both CR-MEGA and MSMA/CA protocols due to its optimized sensing. This is how our HSMA/CA protocol avoids the sensing overhead to spare the fraction of bandwidth for the transmission of more data packets, which ultimately maximizes the system performance. Given the best performance with optimal threshold and sensing interval, it is safe to say that our HSMA/CA is a best candidate MAC protocol for CR-based IoT networks.

## 7. Conclusions

We propose a Handshake Sense Multiple Access with Collision Avoidance (HSMA/CA) mechanism for CR-based IoT networks. Therein, the transmitter first conducts carrier sensing and thereafter it handshakes with the intended receiver using NTS and CTS packets to keep the neighboring SUs silent. Once NTS and CTS are exchanged, the transmitter performs mutual spectrum sensing in conjunction with the receiver in order to detect active PUs and active hidden PUs, respectively. When PU is active, spectrum sensing blocks the transmitter. Otherwise, it waits to receive the ATS packet from the corresponding receiver before transmitting a DATA packet. If the hidden PU is active, then the receiver holds the ATS packet to block the transmitter. Whenever the channel is clear, the transmitter sends an DATA packet and receiver replies with an ACK in the sequel. In case of collision, SUs follow a bakeoff procedure as that in standard CSMA/CA. This is how our MAC protocol protects priority right of PUs by resolving the hidden primary terminal problem, in addition to protecting a densely deployed network from the classical hidden and exposed terminal problems. Our HSMA/CA also adapts the spectrum sensing period by maintaining inefficiencies in spectrum sensing to avoid the sensing overhead. We investigate the performance of the proposed protocol in terms of normalized throughput using the Markov model and duly verified by the simulation. We observe that our HSMA/CA outperforms existing schemes due to optimized spectrum sensing. Hence, the proposed HSMA/CA can be a good candidate MAC protocol in the real environment.

## Figures and Tables

**Table 1:** Summary of the symbols.

Symbol	Description
$\pi_{1,j}$,	Active probability of PU around SU *i*
$\pi_{0,j}$	Inactive probability of PU around SU *i*
$\alpha_{i}$	Probability of false alarm by SU *i*
$\beta_{i}$	Probability of misdetection by SU *i*
*N*	Number of slots in a spectrum sensing period
Ts	Length of a spectrum sensing slot
$P_{i}$	Probability of interference to hidden primary terminals
*A*	Activity factor of hidden primary terminals
*h*	Number of hidden primary terminals
*C*	Capacity of the wireless channel
$R_{i}$	Probability of channel clearance from the active PU at SU *i*
$P_{k}$	Probability of the encountered event $e_{k}$ (k=1,⋯,4)
$t_{k}$	Time delay by encountered event $e_{k}$ (k=1,⋯,4)
$P_{e}$	Probability of empty slots
$P_{t}$	Probability of transmission slots
$P_{n}$	Probability of no-transmission slots
*E*	Transmission rate of the wireless channel
*p*	Probability of a failed transmission
*q*	Probability of a successful transmission
$X_{i}$	Probability of transmission trial by SU *i*
θ	Probability of packet transmission by an SU
*H*	Size of the PHY plus MAC headers
*P*	Size of the packet payload bits an arbitrary SU
σ	Length of an empty (or backoff) slot
θ	Probability of packet transmission by an SU
$W_{0}$	Initial contention window size
*m*	Backoff stage of an arbitrary SU
$W_{m}$	Size of contention window at *m*-th stage
λ, $\hat{\lambda }$	Sensing threshold, Normalized sensing threshold
$\tilde{S}$, S	Throughput, Normalized throughput of HSMA/CA
*M*	Maximum retrial limit of an arbitrary SU
$W_{M}$	Maximum contention window size
$\nu^{2}$, *T*	Noise power, Length of a spectrum sensing period
E[O]	Average length of an arbitrary slot
$E[T_{t}]$	Average time delay of a successful transmission
$E[T_{n}]$	Average time delay of a failed transmission

**Table 2:** Default simulation parameters.

Parameter Name	Value
PHY header	120 bits
MAC header	272 bits
Payload data unit	8184 bits
NTS	160 bits + PHY header
CTS, ATS and ACK	112 bits + PHY header
SIFS time	10 μs
DIFS time	50 μs
Idle slot time	20 μs
Neighbor PU activity rate	0.01
Maximum spectrum sensing time	0.70 ms
Transmission rate	1 Mbps
Initial contention window size ($W_{0}$)	32
Maximum contention window size ($W_{M}$)	1024
Maximum retry limit (*M*)	5

## References

[1] Palattella M.R., Dohler M., Grieco A., Rizzo G., Torsner J., Engel T., Ladid L. (2016). Internet of things in the 5G era: Enablers, architecture, and business models. IEEE J. Sel. Areas Commun..

[2] Evans D. (2011). The Internet of Things: How the Next Evolution of the Internet is Changing Everything.

[3] Whitepaper: IoT Platforms-Enabling the Internet of Things 2016. https://www.ihs.com/Info0416/internet-of-things.html.

[4] (2013). World Population Prospects: The 2012 Revision.

[5] Mitola J., Maguire G.Q. (1999). Cognitive radios: Making software radios more personal. IEEE Pers. Commun..

[6] Ali K.A., Rehmani M.H., Rachedi A. When Cognitive Radio meets the Internet of Things. Proceedings of the 2016 International Wireless Communications and Mobile Computing Conference (IWCMC).

[7] Khan A.A., Rachedi A., Rehmani M.H. (2017). Cognitive-radio-based internet of things: Applications, architectures, spectrum related functionalities, and future research directions. IEEE Wirel. Commun..

[8] Lu W.D., Wang J. (2014). Opportunistic Spectrum Sharing Based on Full-Duplex Cooperative OFDM Relaying. IEEE Commun. Lett..

[9] Lertsinsrubtavee A., Malouch N. (2016). Hybrid Spectrum Sharing through Adaptive Spectrum Handoff and Selection. IEEE Trans. Mob. Comput..

[10] Musavian L., Aissa S. (2009). Fundamental capacity limits of cognitive radio in fading environments with imperfect channel information. IEEE Trans. Commun..

[11] Martinez D.M., Andrade A.G. (2017). Reducing the effects of the noise uncertainty in energy detectors for cognitive radio networks. Int. J. Commun. Syst..

[12] Pandit S., Singh G. (2017). Spectrum Sensing in Cognitive Radio Networks: Potential Challenges and Future Perspective.

[13] Shafiq M., Choi J.-G. (2017). Adaptive Auction Framework for Spectrum Market in Cognitive Radio Networks. J. Netw. Syst. Manag..

[14] Ahmad M., Shafiq M., Irshad A., Afzal M.K., Kim D., Choi J.-G. (2018). Adaptive and Economically-Robust Group Selling of Spectrum Slots for Cognitive Radio-Based Networks. Sensors.

[15] Bentum M.J., Boonstra A.J., Baan W.A. Impact of cognitive radio on radio astronomy. Proceedings of the RFI Mitigation Workshop (RFI 2010).

[16] Ganesan G., Li Y. Agility improvement through cooperative diversity in cognitive radio. Proceedings of the IEEE Global Communications Conference.

[17] Cabric D., Tkachenko A., Brodersen R. Spectrum Sensing Measurements of Pilot, Energy, and Collaborative Detection. Proceedings of the IEEE Military Commun. Conference.

[18] Ganesan G., Li Y. Cooperative spectrum sensing in cognitive radio networks. Proceedings of the First IEEE International Symposium on New Frontiers in Dynamic Spectrum Access Netw.

[19] Su H., Zhang X. (2008). Cross-layer based opportunistic MAC protocols for QoS provisionings over cognitive radio wireless networks. IEEE J. Sel. Areas Commun..

[20] Jeon W.S., Han J.A., Jeong D.G. (2012). A novel MAC scheme for multichannel cognitive radio ad hoc networks. IEEE Trans. Mobile Comput..

[21] Timmers M., Pollin S., Dejonghe A., Perre L.V., Catthoor F. (2009). A distributed multichannel MAC protocol for multihop cognitive radio networks. IEEE Trans. Veh. Technol..

[22] Jha S.C., Phuyal U., Rashid M.M., Bhargava V.K. (2011). Design of OMC-MAC: An opportunistic multi-channel MAC with QoS provisioning for distributed cognitive radio networks. IEEE Trans. Wirel. Commun..

[23] Salameh H.A.B., Krunz M.M., Younis O. (2009). MAC protocol for opportunistic cognitive radio networks with soft guarantees. IEEE Trans. Mob. Comput..

[24] Zhao J., Zheng H., Yang G.-H. Distributed coordination in dynamic spectrum allocation networks. Proceedings of the IEEE Dynamic Spectrum Access Networks (DySPAN 2005).

[25] Brandon L.A. (2011). Survey of common control channel design in cognitive radio networks. Phys. Commun..

[26] Joshi G.P., Nam S.Y., Kim S.W. (2014). Rendezvous Issues in AD Hoc Cognitive Radio Networks. KSII Trans. Internet Inf. Syst..

[27] Huang S., Liu X., Ding Z. Opportunistic spectrum access in cognitive radio networks. Proceedings of the IEEE INFOCOM.

[28] Zhao Q., Tong L., Swami A., Chen Y. (2007). Decentralized cognitive MAC for opportunistic spectrum access in ad hoc networks: A POMDP framework. IEEE J. Sel. Areas Commun..

[29] Adamis A., Constantinou P. Performance study of CSMA/CA over spectrum pooling environment for cognitive radios. Proceedings of the Third IEEE International Conference on Wireless and Mobile Computing, Networking and Communications (WiMob 2007).

[30] Kondareddy Y.R., Agrawal P. Synchronized MAC protocol for multi-hop cognitive radio networks. Proceedings of the 2008 IEEE International Conference on Communications.

[31] Chen Q., Liang Y.-C., Motani M., Wong W.C. (2011). A two-level MAC protocol strategy for opportunistic spectrum access in cognitive radio networks. IEEE Trans. Veh. Technol..

[32] Chen Q., Liang Y.-C., Motani M., Wong W.C. CR-CSMA: A random access mac protocol for cognitive radio networks. Proceedings of the 2009 IEEE 20th International Symposium on Personal, Indoor and Mobile Radio Communications.

[33] Chen Q., Motani M., Liang Y.-C., Wong W.C. Opportunistic spectrum access protocol for cognitive radio networks. Proceedings of the IEEE ICC 2011.

[34] Chen Q., Wong W.C., Motani M., Liang Y.-C. (2013). MAC Protocol Design and Performance Analysis for Random Access Cognitive Radio Networks. IEEE J. Sel. Areas Commun..

[35] Choi Y.-J., Park S., Bahk S. (2006). Multichannel random access in OFDMA wireless networks. IEEE J. Sel. Areas Commu..

[36] Kleinrock L., Tobagi F. (1975). Packet switching in radio channels—Part I: Carrier sense multiple-access modes and their throughput-delay characteristics. IEEE Trans. Commun..

[37] (2007). Wireless LAN Medium Access Control (MAC) and Physical Layer (PHY) Specifications, IEEE Std 802.11-2007 Part 11. https://ieeexplore.ieee.org/servlet/opac?punumber=7786993.

[38] Shafiq M., Son S., Choi J.-G., Yu H. CR-MEGA: Mutually Exclusive Guaranteed Access Control for Cognitive Radio Networks. Proceedings of the IEEE Future Technologies Conference (FTC 2017).

[39] Shafiq M., Choi J.-G., Irshad A. Random Access Control for Cognitive Radio Networks with Hybrid Spectrum Sensing Technique. Proceedings of the International Workshop on Electronics and Commun (WEC 2014).

[40] Shafiq M., Choi J.-G., Yu H., Afzal M.K. Multiple Access Control with Adaptive Spectrum Sensing Technique for Cognitive Radio Networks. Proceedings of the International Workshop on Emerging ICT (JCK-WS 2016).

[41] Arslan H., Yücek T. Spectrum sensing for cognitive radio applications. Proceedings of the Cognitive Radio, Software Defined Radio, and Adaptive Wireless Systems.

[42] Sutton P.D., Nolan K.E., Doyle L.E. (2008). Cyclostationary signatures in practical cognitive radio applications. IEEE J. Sel. Areas Commun..

[43] Shafiq M., Choi J.-G. (2017). MSMA/CA: Multiple Access Control Protocol for Cognitive Radio-Based IoT Networks. J. Internet Technol..

[44] Cabric D., Mishra S.M., Brodersen R.W. Implementation issues in spectrum sensing for cognitive radios. Proceedings of the Signals, Systems and Computers.

[45] Liang Y.-C., Zeng Y., Peh E., Hoang A.T. (2008). Sensing-throughput tradeoff for cognitive radio networks. IEEE Trans. Wire. Commun..

[46] Manesh M.R., Apu M.S., Kaabouch N., Hu W.C. Performance evaluation of spectrum sensing techniques for cognitive radio systems. Proceedings of the IEEE Annual 2016 Ubiquitous Computing, Electronics & Mobile Communication Conference (UEMCON).

[47] Akyildiz I.F., Lee W.-Y., Chowdhury K.R. (2009). CRAHNs: Cognitive radio ad hoc networks. Ad Hoc Netw. J..

[48] Mauwa H., Bagula A., Zennaro M., Pietrosemoli E., Lysko A., Brown T.X. Systematic analysis of Geo-location and spectrum sensing as access methods to TV white space. Proceedings of the IEEE ITU Kaleidoscope: ICTs for a Sustainable World (ITU WT).

[49] Wyglinski M., Nekovee M., Hou T. (2009). Cognitive Radio Communications and Networks: Principles and Practice.

[50] Csurgai-Horvath L., Rieger I., Kertesz J. (2016). A Survey of the DVB-T Spectrum: Opportunities for Cognitive Mobile Users. Mob. Inf. Syst..

[51] Mehdawi M., Riley N., Paulson K., Fanan A., Ammar M. (2013). Spectrum occupancy survey in HULL-UK for cognitive radio applications: Measurement and analysis. Int. J. Sci. Technol. Res..

[52] Patil K., Prasad R., Skouby K. A survey of worldwide spectrum occupancy measurement campaigns for cognitive radio. Proceedings of the International Conference on Devices and Communications (ICDeCom).

[53] Chong J.W., Sung Y., Sung D.K. (2009). RawPEACH: Multiband CSMA/CA-based cognitive radio networks. J. Commun. Netw..

